# MiR-15a-5p Confers Chemoresistance in Acute Myeloid Leukemia by Inhibiting Autophagy Induced by Daunorubicin

**DOI:** 10.3390/ijms22105153

**Published:** 2021-05-13

**Authors:** Emeline Bollaert, Melissa Claus, Virginie Vandewalle, Sandrine Lenglez, Ahmed Essaghir, Jean-Baptiste Demoulin, Violaine Havelange

**Affiliations:** 1 Experimental Medicine Unit, de Duve Institute, Université catholique de Louvain (UCLouvain), Avenue Hippocrate 75 box B1.74.05, 1200 Brussels, Belgium; emeline.bollaert@uclouvain.be (E.B.); meclaus0@gmail.com (M.C.); virginie.vandewalle@uclouvain.be (V.V.); sandrine.lenglez@uclouvain.be (S.L.); ahmed.essaghir@gmail.com (A.E.); jean-baptiste.demoulin@uclouvain.be (J.-B.D.); 2 Department of Hematology, Cliniques Universitaires Saint-Luc, Avenue Hippocrate 10, 1200 Brussels, Belgium

**Keywords:** microRNA, chemoresistance, daunorubicin, autophagy, acute myeloid leukemia, target genes

## Abstract

Anthracyclines remain a cornerstone of induction chemotherapy for acute myeloid leukemia (AML). Refractory or relapsed disease due to chemotherapy resistance is a major obstacle in AML management. MicroRNAs (miRNAs) have been observed to be involved in chemoresistance. We previously observed that *miR-15a-5p* was overexpressed in a subgroup of chemoresistant cytogenetically normal AML patients compared with chemosensitive patients treated with daunorubicin and cytarabine. *MiR-15a-5p* overexpression in AML cells reduced apoptosis induced by both drugs in vitro. This study aimed to elucidate the mechanisms by which *miR-15a-5p* contributes to daunorubicin resistance. We showed that daunorubicin induced autophagy in myeloid cell lines. The inhibition of autophagy reduced cell sensitivity to daunorubicin. The overexpression of *miR-15a-5p* decreased daunorubicin-induced autophagy. Conversely, the downregulation of *miR-15a-5p* increased daunorubicin-induced autophagy. We found that *miR-15a-5p* targeted four genes involved in autophagy, namely *ATG9a, ATG14, GABARAPL1* and *SMPD1*. Daunorubicin increased the expression of these four genes, and *miR-15a-5p* counteracted this regulation. Inhibition experiments with the four target genes showed the functional effect of *miR-15a-5p* on autophagy. In summary, our results indicated that *miR-15a-5p* induces chemoresistance in AML cells through the abrogation of daunorubicin-induced autophagy, suggesting that *miR-15a-5p* could be a promising therapeutic target for chemoresistant AML patients.

## 1. Introduction

Acute myeloid leukemia (AML) is a malignant clonal myeloid neoplasm resulting from the acquisition of several molecular, genetic, and epigenetic alterations in myeloid progenitors, affecting their differentiation, proliferation, and apoptosis [1,2]. The first-line treatment commonly used for de novo AML is the standard induction “7 + 3” chemotherapy regimen combining cytarabine with an anthracycline (daunorubicin or idarubicin). Twenty to thirty percent of young adult patients and fifty percent of older adult patients (≥60 years) with newly diagnosed AML fail to achieve a complete remission due to primary drug resistance or death [3]. In addition, a high percentage of patients who initially achieved a complete remission will relapse due to acquired secondary resistance. Chemotherapy resistance remains the main obstacle in the treatment of AML and critically contributes to short-term overall survival. The mechanisms of drug resistance in AML are poorly understood. A main research challenge in AML therapy is the identification of pathways involved in chemotherapy resistance that could provide new targets for future therapeutic interventions.

Daunorubicin (DNR) is the most widely used anthracycline combined with cytarabine in the standard “7 + 3” induction regimen. DNR dose intensification has been an important topic in the last decade. Clinical trials reported improvements in remission rates as well as in survival with higher doses of DNR in subgroups of AML patients following cytogenetic and molecular classification [4,5]. Patients younger than 65 years old or with an unfavorable cytogenetic risk seemed to benefit from high doses of DNR without increased toxicity [6]. A deeper understanding of the mechanisms of DNR resistance will help to choose the best therapy and anthracycline dose for each AML patient without additional toxicities.

Autophagy is an important homeostatic cellular recycling program. In cancer cells, autophagy constitutes a prosurvival and cytoprotective mechanism in response to stress conditions but can also, if inappropriately activated, promote apoptosis and/or autophagic programmed cell death. In response to a wide array of therapies, autophagy in human AML cells predominantly drives a cytodestructive cascade that induces the catabolism of oncogenic fusion proteins and leads to cell death. Arsenic trioxide, a potent inducer of autophagy in AML, induced anti-leukemic effects via p62/SQSTM1-mediated degradation of the PML/RARA oncoprotein in NB4 cells [7]. Similarly, autophagy induction by proteasome inhibitors was responsible for the degradation of FLT3-ITD and subsequent activation of cell death in AML cell lines [8]. Furthermore, several studies demonstrated a decrease in autophagy levels in primary human blast cells and that a loss of key autophagy genes leads to leukemia initiation and progression [9,10]. Multiple autophagy genes were localized within chromosomal regions commonly heterozygously deleted in AML, including *ATG12, GABARAPL1*, and *GABARAPL2*, whose expressions were particularly affected in AML blasts [9].

MicroRNAs (miRNAs) constitute a family of small non-coding RNAs (18–24 bp) that bind to the 3′ untranslated region (UTR) of target transcripts to induce their degradation or block their translation. Accumulating evidence suggests that miRNAs play critical roles in AML by regulating the expression of a wide array of target genes involved in cell survival, proliferation, differentiation, and apoptosis [11,12]. In addition, miRNAs are important regulators of autophagy [13,14,15]. For instance, *miR-130a* targeted ATG2B to inhibit autophagy and promote chronic lymphocytic leukemia cell death [14]. In chronic myeloid leukemia cells, *miR-30a* increased imatinib sensitivity through the regulation of ATG5 and *BECN1*/Beclin 1 [16]. In AML cells, the overexpression of *miR-106a* prevented ULK1 induction by all-trans retinoic acid (ATRA) treatment [10].

A wide range of studies has demonstrated the role of multiple miRNAs in modulating the sensitivity to cytotoxic agents in AML cell lines [17,18,19,20]. However, little is known about the roles of miRNAs in chemoresistance in AML patients. In our previous study, we showed that *miR-21-5p* and *miR-15a-5p* were overexpressed in a subgroup of chemoresistant, cytogenetically normal AML patients compared to chemosensitive patients treated with the standard induction chemotherapy combining cytarabine and DNR [21]. In AML patients, a high expression of *miR-15a-5p* was shown to predict shorter survival and worse response to chemotherapy [22]. The mechanisms of chemoresistance induced by *miR-15a-5p* in AML are not completely understood.

In the current study, we assessed the role of *miR-15a-5p* in DNR-induced autophagy. We identified and validated four target genes involved in autophagy. Our data provide a new mechanism of DNR resistance in AML by which *miR-15a-5p* negatively regulates chemotherapy-induced cytotoxic autophagy.

## 2. Results

### 2.1. Daunorubicin Induces Autophagy in Myeloid Cell Lines

**Figure 1:**
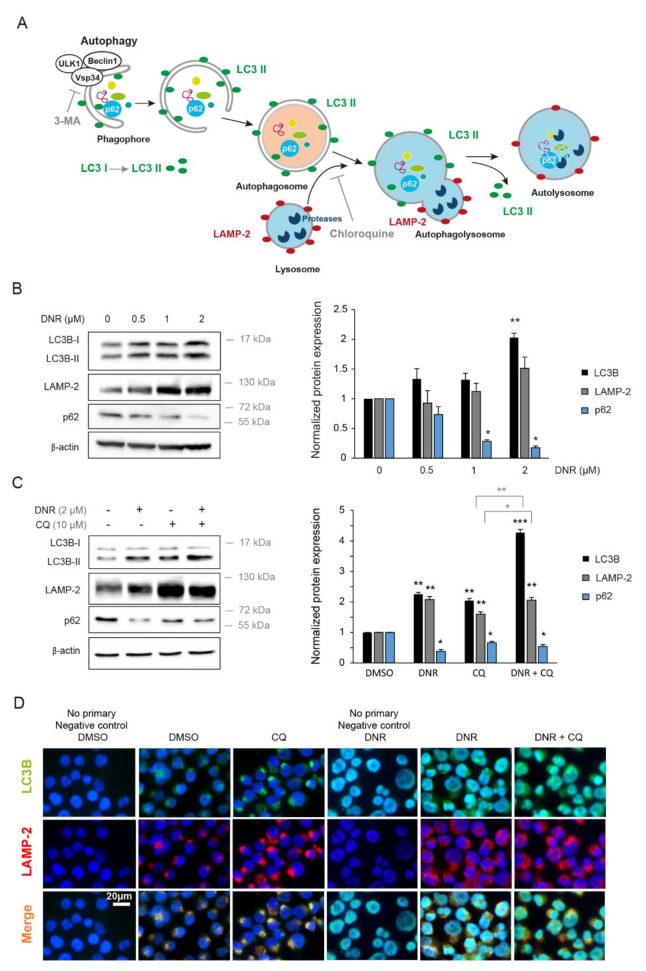
Treatment with daunorubicin increases autophagy in K562 cells. (**A**) Schematic representation of the autophagy process with its components and related molecules. (**B**) K562 cells were treated with different indicated doses of daunorubicin (DNR) or vehicle (DMSO) for 24 h. (**C**,**D**) K562 cells were treated with 2 µM of daunorubicin for 24 h and 10 µM of chloroquine (CQ) for the last 4 h. (**B**,**C**) Level of autophagy was analyzed by Western blotting with anti-LC3B, anti-LAMP-2, and anti-p62 antibodies. Expression of β-actin was also detected as a loading control. The Western blotting band intensities of three independent experiments were quantified using the ImageJ software. The untreated conditions were set to 1. The normalized means are shown with SEM. (*, *p* < 0.05, **, *p* < 0.01, ***, *p* < 0.001, in black: all the conditions compared with the untreated conditions, and in grey: CQ conditions compared with DNR and CQ conditions). (**D**) Double-labeling immunofluorescence and representative images of LC3 (in green) and LAMP-2 (in red). Nuclear staining (DAPI, blue) is also shown.

The autophagy process begins with the formation of an isolation membrane known as the phagophore. The assembly of the phagophore is promoted by conditions of nutrient, energy, or growth factor deprivation, and requires the conjugation of microtubule-associated protein 1 light chain 3 (LC3). ULK1 turns on the subsequent vesicle nucleation by regulating the activation of a multiprotein complex composed in part of Beclin 1 and the class III phosphoinositide 3-kinase (PI3K) Vsp34. Portions of cytoplasm, protein aggregates, or organelles are sequestered within double-membraned vesicles called autophagosomes. The initiation and elongation of autophagosomes are mediated by multiple members of the autophagy gene (ATG) family. Subsequently, loaded autophagosomes fuse with lysosomes to form autolysosomes, enabling cellular cargo degradation and recycling (Figure 1A) [13,23].

To evaluate the regulation of autophagy by daunorubicin (DNR), we first analyzed the conversion of the cytosolic form of LC3 (LC3-I) to the conjugated form (LC3-II), which is recruited to autophagosomal membranes (Figure 1A), by Western blotting. High doses of DNR (2 µM) enabled the switch of LC3-I into LC3-II and were accompanied with a significant increased expression level of LC3 in DNR-treated myeloid leukemia cell lines K562 and KG1a (Figure 1B and Figure S1A). Consistently, the expression of LAMP-2, a lysosomal membrane protein involved in chaperone-mediated autophagy (Figure 1A), was up-regulated in AML cells treated with DNR (Figure 1B and Figure S1A). Concurrently, p62/SQSTM1, an intra-autophagosomal component that is degraded by autophagy (Figure 1A), was significantly downregulated by DNR treatment. The effects of DNR on these proteins were dose-dependent (Figure 1B and Figure S1A).

We next used chloroquine (CQ), a lysosomal inhibitor that impairs autophagosome fusion with lysosomes (Figure 1A), to monitor autophagic flux in the presence of DNR. As expected, CQ treatment triggered the accumulation of LC3 and LAMP-2 autophagic markers, as shown by Western blotting and immunofluorescence (Figure 1C,D). In combination with CQ, DNR significantly increased the expression of LC3-II and LAMP-2 and decreased p62/SQSTM1 compared with the vehicle condition, indicating that DNR activated an autophagic flux (Figure 1C). Moreover, the treatment of K562 cells with DNR increased the immunofluorescence staining of LC3, which localizes to isolation membranes and autophagosomes, and the staining of LAMP-2, a marker for autophagosomes and lysosomes (Figure 1D). Noticeably, without treatment, the LC3^+^/LAMP-2^+^ staining highlighted autophagosomes, which appeared as perinuclear punctuations, whereas under DNR treatment, the staining distribution was more diffuse in the cells (Figure 1D). Taken together, our results show that autophagy is promoted by DNR in myeloid leukemia cell lines, in accordance with the study of Han et al. [24].

### 2.2. Autophagy Inhibition Increases Myeloid Cell Growth and Prevents Downregulation by Daunorubicin

**Figure 2:**
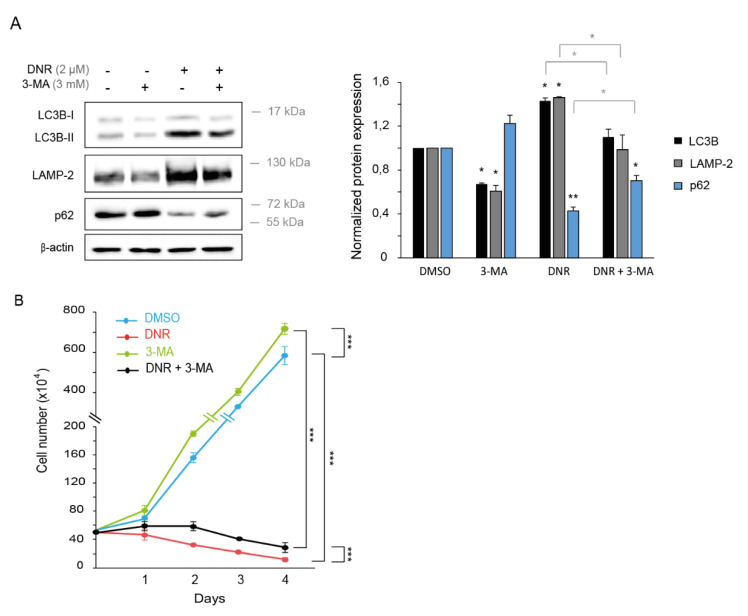
Treatment with 3-MA partially prevents the effects of DNR on autophagy and cell growth. K562 were treated with 2 µM of daunorubicin and 3 mM of 3-methyladenine (3-MA). (**A**) Proteins were extracted after 24 h, and the level of autophagy was analyzed by Western blotting with anti-LC3B, anti-LAMP-2, and anti-p62 antibodies. Expression of β-actin was also detected as a loading control. The Western blotting band intensities of three independent experiments were quantified using the ImageJ software. The untreated conditions were set to 1. The normalized means are shown with SEM. (*, *p* < 0.05, **, *p* < 0.01, in black: all the conditions compared with the untreated conditions, and in grey: DNR conditions compared with DNR and 3-MA conditions). (**B**) Viable cells were counted in the presence of Trypan Blue after 1, 2, 3, and 4 days of culture. The mean of three independent experiments is shown with SEM. The statistical analysis was by a two-way ANOVA followed by Bonferroni’s test (***, *p* < 0.001).

Next, we assessed the implication of autophagy regulation by DNR in myeloid leukemia cell growth. To prevent the induction of autophagy by DNR, we treated K562 cells with 3-methyladenine (3-MA), which blocks autophagosome formation via the inhibition of class III phosphatidylinositol-3 kinase (PI3K) (Figure 1A). As expected, autophagy inhibition by 3-MA resulted in a decreased protein level of LC3-II and LAMP-2 and an increased expression of p62/SQSTM1 (Figure 2A). In addition, 3-MA treatment partially abolished the effects of DNR on autophagy, as shown by Western blotting (Figure 2A).

We analyzed the survival of K562 cells by counting the number of cells over four days in the presence of DNR and/or 3-MA treatment. First, we observed that DNR totally prevented K562 cell proliferation and that 3-MA increased cell growth (Figure 2B). Second, the combination of 3-MA and DNR significantly increased K562 cell survival compared with the DNR alone condition (Figure 2B), suggesting that DNR decreases myeloid leukemia cell growth partially by inducing autophagy.

### 2.3. Overexpression of miR-15a-5p Inhibits Autophagy Induced by Daunorubicin

**Figure 3:**
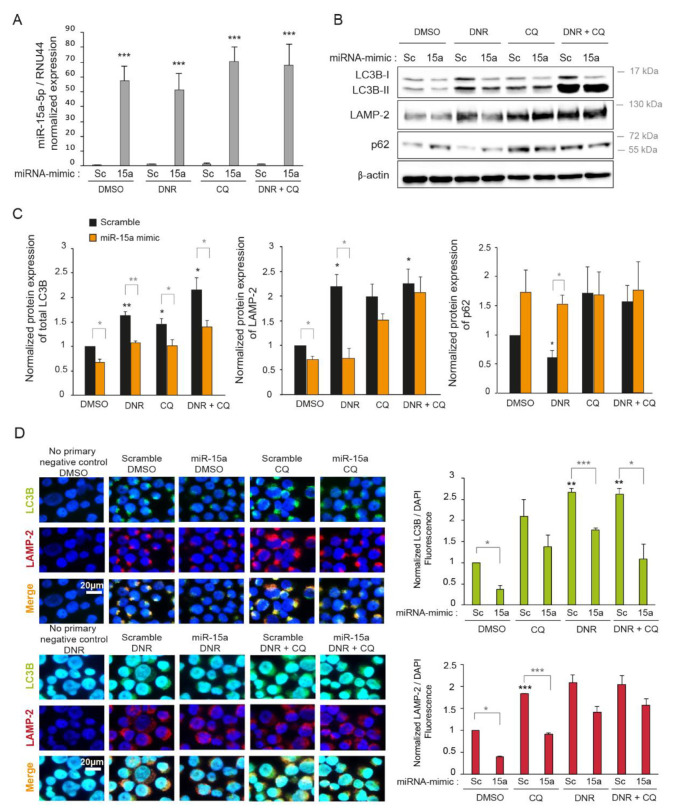
*miR-15a-5p* inhibits autophagy induced by daunorubicin. K562 cells were transiently transfected with miR-15a-5p mimic (15a) or scrambled mimic (Sc) as control for 48 h, and cells were treated with 2 µM of daunorubicin (DNR) for the last 24 h and with 10 µM of chloroquine (CQ) for the last 16 h. (**A**) RNA was extracted and *miR-15a-5p* expression was measured by RT-qPCR. Normalization was completed with the endogenous control *RNU44*. (**B**) Proteins were extracted, and the level of autophagy was analyzed by Western blotting with anti-LC3B, anti-LAMP-2, and anti-p62 antibodies. Expression of β-actin was also detected as a loading control. (**C**) The Western blotting band intensities of three independent experiments were quantified using the ImageJ software. (**D**) Double-labeling immunofluorescence and representative images of LC3 (in green) and LAMP-2 (in red). Nuclear staining (DAPI, blue) is also shown. LC3B and LAMP-2 immunofluorescence intensities of three independent experiments were quantified using the ImageJ software and divided by the nuclear immunofluorescence. (**A**,**C**,**D**) The untreated scrambled condition was set to 1. The normalized means of three independent experiments are shown with SEM. (*, *p* < 0.05, **, *p* < 0.01, ***, *p* < 0.001, in black: all the conditions compared with the untreated scrambled condition, and in grey: miR-15a conditions compared with scrambled conditions).

In our previous study, we found that *miR-15a-5p* was significantly overexpressed in a subgroup of cytogenetically normal chemoresistant AML patients compared with chemosensitive patients [21]. In addition, we showed that *miR-15a-5p* contributed to chemoresistance by reducing apoptosis induced by cytarabine and DNR [21]. Interestingly, using microarray analysis on K562 cell line treated with DNR (1 µM), we found that *miR-15a-5p* could downregulate not only pro-apoptotic genes but also genes involved in autophagy (Figure S2). This result suggested that the overexpression of *miR-15a-5p* in AML could also induce resistance to DNR treatment by regulating autophagy. To test whether *miR-15a-5p* was able to regulate autophagy, we overexpressed an *miR-15a-5p* mimic in myeloid leukemia K562 and KG1a cell lines treated with 2 µM of DNR or a vehicle and analyzed the expression of autophagy markers. The efficiency of transfection was confirmed by measuring the expression of mature *miR-15a-5p* using RT-qPCR. *MiR-15a-5p* expression was increased by about 50 times and 3 times upon transfection of K562 and KG1a cells, respectively (Figure 3A and Figure S1B). Remarkably, the DNR treatment did not modify *miR-15a-5p* expression before or after transfection (Figure 3A and Figure S1B). As shown in Figure 3B,C and Figure S1B, *miR-15a-5p* overexpression resulted in significantly decreased protein levels of LC3 and LAMP-2 and a concurrent increase of p62/SQSTM1 in cells treated with or without DNR, suggesting that *miR-15a-5p* might negatively regulate autophagy. To confirm the downregulation of autophagy by *miR-15a-5p*, we also assessed autophagosome formation by immunofluorescence. Cells transfected with *miR-15a-5p* demonstrated a decrease of both LC3 and LAMP-2 staining compared with the scrambled condition (Figure 3D). Interestingly, the overexpression of *miR-15a-5p* also prevented in part the autophagosome formation induced by DNR and was able to limit the accumulation of autophagosomes under CQ treatment (Figure 3D), indicating that *miR-15a-5p* expression reduced the autophagic flux. Collectively, our results showed that *miR-15a-5p* expression inhibits basal level and DNR-induced autophagy in myeloid leukemia cell lines.

### 2.4. Overexpression of miR-15a-5p Increases Myeloid Cell Growth and Prevents Downregulation by Daunorubicin

**Figure 4:**
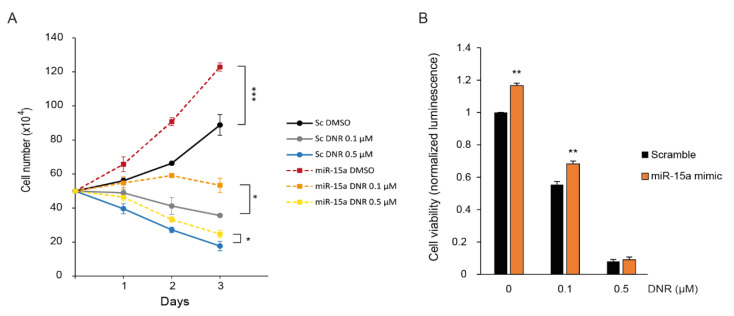
*miR-15a-5p* decreases the downregulation of cell growth by daunorubicin. K562 cells were transiently transfected with miR-15a-5p mimic (miR-15a) or scrambled mimic (Sc) as control, and cells were treated with 0.1 µM of daunorubicin (DNR), 0.5 µM of DNR, or vehicle for 72 h (**A**) Viable cells were counted in the presence of Trypan Blue after 1, 2, and 3 days of culture. The mean of three independent experiments is shown with SEM. The statistical analysis was by a two-way ANOVA followed by Bonferroni’s test (*, *p* < 0.05, ***, *p* < 0.001). (**B**) Cell viability assay (CellTiter-Glo^®^ Luminescent assay) was performed at 72 h of treatment. The untreated scrambled condition was set to 1. The normalized means are shown with SEM (**, *p* < 0.01).

Then, we assessed the regulation of myeloid leukemia cell growth by *miR-15a-5p* under DNR treatment. We analyzed the survival of K562 cells transfected with a *miR-15a-5p* mimic by counting the number of cells over three days in the presence of 0.1 µM of DNR, 0.5 µM of DNR, or vehicle. We also performed a cell viability assay after 72 h of DNR treatment. First, we observed that the overexpression of *miR-15a-5p* significantly increased K562 cell growth compared with the scrambled condition (Figure 4A,B). Second, *miR-15a-5p* overexpression significantly prevented the downregulation of cell survival upon DNR treatment (Figure 4A,B), suggesting that *miR-15a-5p* decreases the DNR induction of autophagic cell death.

### 2.5. Inhibition of miR-15a-5p Induces Autophagy and Decreases Myeloid Cell Growth

**Figure 5:**
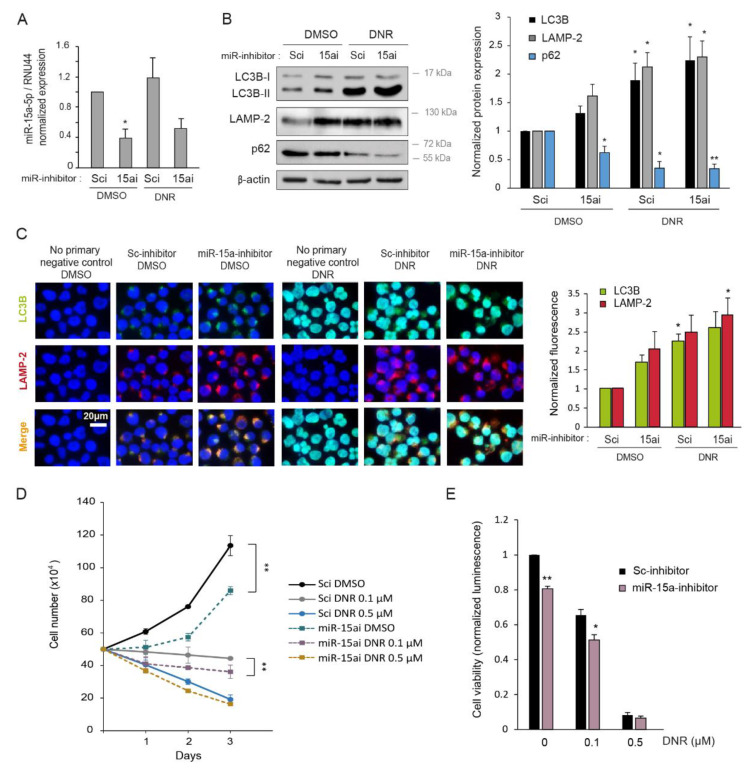
Inhibition of *mir-15a-5p* induces autophagy. (**A**–**C**) K562 cells were transiently transfected with *miR-15a-5p* inhibitor (15ai) or scrambled inhibitor (Sci) as control for 48 h, and cells were treated with 2 µM of daunorubicin (DNR) for the last 24 h. (**A**) RNA was extracted and *miR-15a-5p* expression was measured by RT-qPCR and normalized with the expression of *RNU44*. (**B**) Protein were extracted, and level of autophagy was analyzed by Western blot with anti-LC3B, anti-LAMP-2, and anti-p62 antibodies. Expression of β-actin was also detected as a loading control. (**C**) The Western blotting band intensities of three independent experiments were quantified using the ImageJ software. (**D**) Double-labeling immunofluorescence and representative images of LC3B (in green) and LAMP-2 (in red). Nuclear staining (DAPI, blue) is also shown. LC3B and LAMP-2 immunofluorescence intensities of three independent experiments were quantified using the ImageJ software and divided by the nuclear immunofluorescence. (**D**,**E**) K562 cells were transiently transfected with miR-15a-5p inhibitor (miR-15ai) or scrambled inhibitor (Sci) as control, and cells were treated with 0.1 µM of daunorubicin (DNR), 0.5 µM of DNR, or vehicle for 72 h. (**D**) Viable cells were counted in the presence of Trypan Blue after 1, 2, and 3 days of culture. (**E**) Cell viability assay (CellTiter-Glo^®^ Luminescent assay) was performed at 72 h of treatment. (**A**–**C**,**E**) The untreated scrambled conditions were set to 1. The normalized means of three independent experiments are shown with SEM. (*, *p* < 0.05, **, *p* < 0.01, all the conditions compared with the untreated scrambled condition). (**D**) The mean of three independent experiments is shown with SEM. The statistical analysis was by a two-way ANOVA followed by Bonferroni’s test (**, *p* < 0.01).

To further evaluate the regulation of autophagy by *miR-15a-5p*, we tested the effect of *miR-15a-5p* inhibition. For this purpose, K562 and KG1a myeloid cell lines were transfected with *miR-15a-5p* mirVana inhibitor versus a scrambled control, and cells were treated with or without 2 µM of DNR. Inhibition of *miR-15a-5p* was confirmed by RT-qPCR and resulted in a significant decrease of *miR-15a-5p* expression (Figure 5A and Figure S1C). As shown in Figure 5B,C and Figure S1C, myeloid leukemia cells transfected with *miR-15a-5p* inhibitor revealed increased amounts of LC3-II and LAMP-2 detected by Western blotting and immunofluorescence and a decreased protein level of p62/SQSTM1 relative to the scrambled condition. This result confirmed the negative regulation of autophagy by *miR-15a-5p*. Moreover, the inhibition of *miR-15a-5p* recapitulated the autophagy regulation obtained with DNR.

We also analyzed the proliferation and viability of K562 cells transfected with miR-15a-5p inhibitor upon DNR treatment. We observed that the inhibition of *miR-15a-5p* significantly decreased K562 cell growth compared with the scrambled condition (Figure 5D,E). In addition, *miR-15a-5p* inhibition enhanced the decrease of myeloid cell viability induced by DNR, as shown by cell counting and cell viability assay (Figure 5D,E).

### 2.6. MiR-15a-5p Downregulates the Expression of Autophagy Target Genes

We next investigated potential target genes involved in the regulation of autophagy by *miR-15a-5p* and in chemoresistance. Our microarray analysis showed that, of the transcripts downregulated by *miR-15a-5p* in K562 cells treated with DNR, four were implicated in autophagy: *ATG9A*, *ATG14*, *GABARAPL1,* and *SMPD1* (Figure S2A–D). This list was crossed in advance with target mRNAs identified by the TargetScan prediction program [25] (Figure S2A), revealing that the 3′UTR region of these four transcripts contained one or two predicted binding sites for *miR-15a-5p* (Figure S2E). ATG9a, which is an autophagy-related membrane protein localized in the phagophore/pre-autophagosomal structure, plays a crucial role in the formation of autophagosomes [26]. ATG14 forms a protein complex with Beclin 1, Vsp15, and Vsp34 (the catalytic subunit of class III PI3K), which initiates autophagy [27]. In addition, ATG14 promotes autophagosome–endolysosome fusion by interacting with the STX17–SNAP29 binary t-SNARE complex. GABARAPL1 is required for the late maturation of autophagosomes [28]. Finally, *SMPD1* encodes a lysosomal acid sphingomyelinase also known as ASM and participates in the late autophagy stage associated with the lysosomes [29].

**Figure 6:**
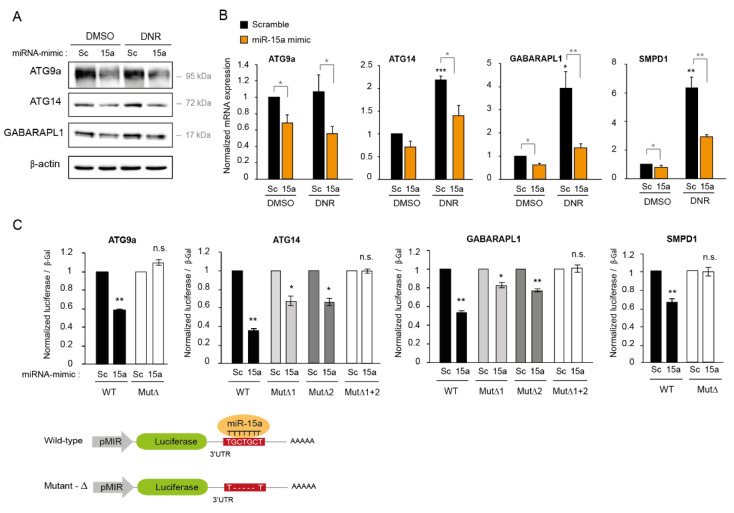
*miR-15a-5p* directly regulates the expression of autophagy target genes. (**A**,**B**) K562 cells were transiently transfected with *miR-15a-5p* mimic (15a) or scrambled mimic (Sc) as control and treated with 2 µM of daunorubicin (DNR) for 24 h before protein and RNA extraction. (**A**) Protein expression of ATG9A, ATG14, and GABARAPL1 was analyzed by Western blotting. Expression of β-actin was also detected as a loading control. (**B**) The expression of *ATG9A, ATG14, GABARAPL1,* and *SMPD1* was measured by RT-qPCR and normalized with the expression of a housekeeping gene *RPLP0.* (**C**) HEK 293T cells were co-transfected with wild-type (WT) or mutated (Mut∆) pMIR-luciferase vector, with either *miR-15a-5p* mimic or the scrambled oligonucleotide as control, and the pEF-β-galactosidase vector as internal control. After 24 h of transfection, cells were lysed, and the luminescence and β-gal activities were measured. (**B**,**C**) The normalized means of three independent experiments are shown with SEM. The scrambled condition was set to 1. (*, *p* < 0.05, **, *p* < 0.01, ***, *p* < 0.001, in black: all the conditions compared with the scrambled condition, and in grey: miR-15a conditions compared with scrambled conditions, n.s.: non-significant).

To validate the regulation of these four potential targets by *miR-15a-5p*, we measured their expression by Western blotting and RT-qPCR after the ectopic expression of *miR-15a-5p* in K562 cells. The overexpression of *miR-15a-5p* resulted in a downregulation of *ATG9A, ATG14,* and *GABARAPL1* at protein and RNA levels with respect to the scrambled condition in untreated cells and in cells treated with DNR (Figure 6A,B). *SMPD1* expression was also reduced by *miR-15a-5p*. This was only validated at the RNA level because we were not able to detect *SMPD1* expression by Western blotting due to a lack of functional antibodies (Figure 6B). Interestingly, DNR treatment significantly increased the expression of these autophagy genes at the protein and/or RNA level, except for *ATG9A*, and the overexpression of *miR-15a-5p* counteracted this induction (Figure 6A,B). To confirm the direct regulation of these four targets by *miR-15a-5p*, we cloned the 3′UTR regions of *ATG9A, ATG14, GABARAPL1,* and *SMPD1*, which were predicted by TargetScan to interact with *miR-15a-5p* into a pMIR-luciferase reporter (Figure 6C and Figure S2E). There was one predicted interaction site for *miR-15a-5p* in the *ATG9A* and *SMPD1* 3′UTR and two interaction sites in the *ATG14* and *GABARAPL1* 3′UTR (Figure S2E). We co-transfected the luciferase reporter with the synthetic *miR-15a-5p* into HEK 293T cells. We observed a significant reduction (40% to 60%) in the luciferase activity for these four target constructs transfected with *miR-15a-5p* (Figure 6C). This effect was abrogated when we co-transfected mutated luciferase reporter vectors carrying deletions of five bases in the seed sequences of *miR-15a-5p* (Figure 6C). For *ATG9A* and *SMPD1*, the mutation of the only predicted site was sufficient to lose the direct regulation by *miR-15a-5p*. For *ATG14* and *GABARAPL1*, which contain two predicted binding sites for *miR-15a-5p*, the mutation of one site had a partial effect. Nevertheless, the double mutant abrogated the decrease in luciferase activity, indicating that the two 3′UTR binding sites were necessary for the regulation of *ATG14* and *GABARAPL1* by *miR-15a-5p*. Altogether, these experiments showed that *miR-15a-5p* directly regulated the expression of four autophagy genes: *ATG9A, ATG14, GABARAPL1,* and *SMPD1*.

### 2.7. Expression of ATG9A, ATG14, and GABARAPL1 Are Downregulated in Chemoresistant AML Patients

**Figure 7:**
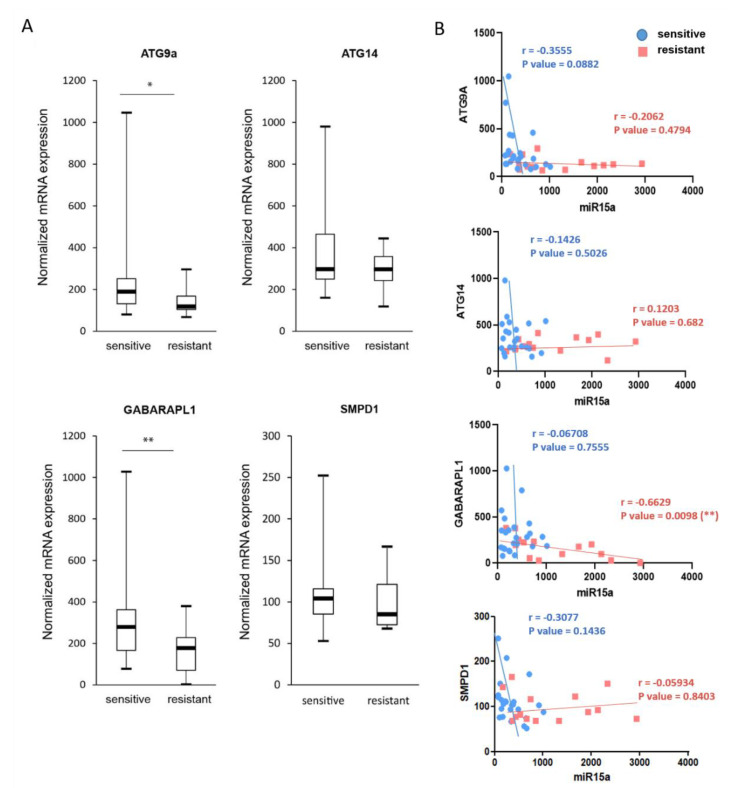
*Expression of ATG9A, ATG14, GABARAPL1, and SMPD1 in AML patient samples*. (**A**) Expression of *ATG9A, ATG14, GABARAPL1,* and *SMPD1* was analyzed by gene expression profiles (Affymetrix) in AML patients with a normal karyotype or who were sensitive (*n* = 24) or resistant (*n* = 14) to standard chemotherapy treatment combining cytarabine and daunorubicin, available in the HOVON dataset (GEO accession: GSE6891). The box plot is based on microarray normalized mRNA expression. The statistical analysis was obtained using Wilcoxon test (*, *p* < 0.05, **, *p* < 0.01). (**B**) The correlation plot of *miR-15a-5p* and gene expression in AML patients is shown with Pearson**** correlation coefficient (Pearson’s r) and *p* values for sensitive (in blue) and resistant (in pink) patients.

To assess the implication of autophagy regulation by *miR-15a-5p* in chemoresistance, we analyzed the gene expression profiles from the HOVON dataset collected with a cohort of AML patient samples, as previously described (GEO accession: GSE6891) [30]. Gene expression was measured in bone marrow samples from 38 AML patients at diagnosis before treatment with standard chemotherapy by using Affymetrix U133A GeneChips. We selected samples from patients younger than 65 years with cytogenetically normal AML and wild-type *NPM1* without *FLT3*-ITD. We compared the four target genes *ATG9A*, *ATG14*, *GABARAPL1,* and *SMPD1* expression in chemosensitive (n = 24) and chemoresistant (n = 14) patients. Figure 7A shows the downregulation of the four target genes in chemoresistant AML patients compared with chemosensitive patients, with a significant differential expression for *ATG9A* and *GABARAPL1* between both groups of patients, confirming the direct roles of the autophagy targets in chemoresistance. The expression of *miR-15a-5p* was previously analyzed in this cohort of AML patients by small RNA-sequencing, and we reported a significant overexpression of *miR-15a-5p* in chemoresistant compared with chemosensitive AML patients [21]. Figure 7B shows the correlation plot of *miR-15a-5p* and autophagy gene expression. We observed a negative correlation between *miR-15a-5p* and *ATG9A, ATG14*, *GABARAPL1,* and *SMPD1* gene expression in chemosensitive and chemoresistant patients, suggesting that the overexpression of *miR-15a-5p* observed in chemoresistant AML patients contributes to chemoresistance by reducing the expression of autophagy target genes.

### 2.8. Downregulation of miR-15a-5p Targets Decreases the Activation of Autophagy

**Figure 8:**
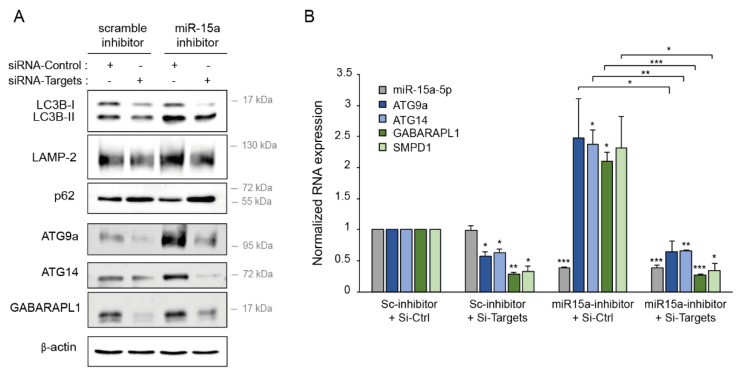
*miR-15a-5p* regulates autophagy by targeting autophagy genes. K562 cells were transiently co-transfected with *miR-15a-5p* inhibitor or scrambled inhibitor as control and with either a combination of four siRNA-targets (*ATG9A, ATG14, GABARAPL1,* and *SMPD1*) or a siRNA-control for 24 h before protein and RNA extractions. (**A**) Level of autophagy was analyzed by Western blotting with anti-LC3B, anti-LAMP-2, and anti-p62 antibodies. Protein expression of ATG9A, ATG14, and GABARAPL1 was also analyzed, as well as the expression of β-actin as a loading control. (**B**) In all the conditions, the expression of *miR-15a-5p, ATG9A, ATG14, GABARAPL1,* and *SMPD1* was measured by RT-qPCR and normalized with the expression of *RNU44* for *miR-15a-5p* or with *RPLP0* for the autophagy target genes. The scrambled inhibitor and siRNA condition was set to 1. The normalized means of three independent experiments are shown with SEM. (*, *p* < 0.05, **, *p* < 0.01, ***, *p* < 0.001, all the conditions compared with the scrambled condition).

We next determined whether the autophagy targets of *miR-15a-5p* participated in its regulation of autophagy using siRNAs against *ATG9A, ATG14, GABARAPL1,* and *SMPD1* to downregulate their expression. To assess the impact on autophagy, we co-transfected K562 cells with a miR-15a-5p inhibitor to increase the autophagy process and with a combination of siRNAs against each target gene. The efficiency of siRNAs was evaluated by Western blotting and RT-qPCR, demonstrating a significant downregulation of *ATG9A, ATG14, GABARAPL1,* and *SMPD1* expression at protein and/or mRNA levels (Figure 8A,B). Moreover, the inhibition of *miR-15a-5p* led to the overexpression of the four targets, confirming their direct regulation. As expected, the combined decreased expression of *ATG9A, ATG14, GABARAPL1,* and *SMPD1* reduced basal autophagy, as shown by the reduced expression of LC3 and LAMP-2 protein and increased expression of p62/SQSTM1 protein (Figure 8A). The downregulation of the four target genes indeed reproduced the functional effects of *miR-15a-5p*. Finally, we observed that the combined downregulation of *ATG9A, ATG14, GABARAPL1,* and *SMPD1* using siRNAs blocked autophagy induced by inhibition of *miR-15a-5p*. Collectively, these results showed that *miR-15a-5p* regulated autophagy by directly targeting several genes involved in different steps of this process.

In conclusion, we demonstrated that the direct regulation of *ATG9A, ATG14, GABARAPL1,* and *SMPD1* contributes to the inhibition of autophagy by *miR-15a-5p*.

## 3. Discussion

Acute myeloid leukemia still has in most cases a poor long-term survival rate with a high risk of relapse due to resistance to the standard induction chemotherapy combining cytarabine and DNR. This paper provides new evidence for the important role of miRNAs in DNR resistance in AML mediated by target genes implicated in autophagy.

The induction of autophagy in response to several chemotherapies can favor either death or survival, contributing to drug efficacy or resistance [31]. Most chemotherapies activate autophagy in cancer cells as a protective response to stress-induced damage [23]. Emerging studies show that chemotherapies can also induce autophagy cell death. In our study, we demonstrated that high doses of DNR induced autophagy in myeloid leukemia cell lines, as previously reported by Han and colleagues [24]. Treatment of K562 cells with DNR enables LC3-I/II conversion, punctate distribution of endogenous LC3, and p62 degradation. We also found that DNR-induced autophagy plays a pivotal role in leukemic cell survival. Indeed, the DNR chemosensitivity was partially prevented by pre-treating cells with 3-MA, an inhibitor of autophagy. Similarly, Ristic et al. demonstrated that idarubicin induces cytotoxic autophagy in K562 cell line [32]. Autophagy inhibitors reduced the cytotoxicity of idarubicin in K562 cells. Idarubicin-induced autophagy contributes to the proapoptotic action of chemotherapy [32]. We were not able to observe an induction of autophagy after cytarabine treatment of K562 cells at low or high doses (data not shown).

In agreement with our observations, Jin and colleagues showed that autophagy was reduced in AML blasts, and key autophagy genes such as *ULK1*, *ATG3*, *ATG4D,* and *ATG5* were found to be downregulated in primary AML patient samples [10]. Watson et al. concluded that decreased autophagy may facilitate aberrant proliferation and contribute to AML development [9]. Accordingly, we observed an increase of K562 cell growth after treatment with the 3-MA autophagy inhibitor. We also found that *ATG9A*, *ATG14*, *GABARAPL1,* and *SMPD1* gene expression was downregulated in chemoresistant AML patient samples compared with chemosensitive patients. Despite its dual role in cancer, autophagy has been robustly shown to kill acute myeloid leukemia cells, especially via the degradation of the oncogenic fusion protein that drives leukemogenesis [33]. For instance, the anti-leukemic role of autophagy has been reported in AML with FLT3-ITD alteration. Larrue et al. showed that FLT3-ITD molecules become detectable within the autophagosomes and are eventually degraded upon proteasome inhibitor-initiated autophagy [8].

Our previous studies investigated the important roles of miRNAs in mediating sensitivity and resistance to chemotherapeutic agents in AML. We previously demonstrated that *miR-15a-5p* decreased apoptosis induced by DNR and/or cytarabine in leukemia by downregulating three pro-apoptotic target genes—*PDCD4*, *ARL2,* and *BTG2*—validating the implication of *miR-15a-5p* in drug resistance [21]. Our results revealed that *miR-15a-5p* contributes to leukemia chemotherapy resistance by regulating, in addition to apoptosis, autophagy induced by DNR treatment. Accordingly, we observed an increase of K562 cell growth after the overexpression of *miR-15a-5p* and a decrease of cell growth after the inhibition of *miR-15a-5p*. Our gain-of-function experiments in AML cell lines showed that *miR-15a-5p* overexpression decreased the activation of autophagy and the downregulation of K562 cell viability after DNR treatment.

To date, several miRNAs have been associated with autophagy in myeloid leukemia: *miR-21*, *miR-30a, miR-34a,* and *miR-125b-1* [13,17]. In K562 and KYO-1 cells, *miR-21* downregulation increased the expression of the autophagy-related proteins Beclin-1, Vsp34, and LC3-II and sensitized leukemic cells to doxorubicin [34]. Yu and colleagues described *miR-30a* as a potent inhibitor of autophagy whose expression is inversely correlated with Beclin-1 and ATG5 in K562 cells, enhancing imatinib-induced cytotoxicity [16]. A higher expression level of *miR-34a* suppresses all-trans retinoic acid (ATRA)-induced autophagy in HL60 cell line by stimulating LC3 conversion [35]. *miR-125b-1* was reported to be highly expressed in patients with acute promyelocytic leukemia [36]. The overexpression of *miR-125b-1* inhibited the autophagy–lysosomal pathway induced by ATRA and impaired PML-RARA degradation. This impairment subsequently arrests cell differentiation [36].

In the present study, we demonstrated that *miR-15a-5p* prevented basal and DNR-induced autophagy in AML by directly downregulating four genes involved in this catalytic process: *ATG9A, GABARAPL1, ATG14,* and *SMPD1.* Thereby, *miR-15a-5p* might prevent autophagy by inhibiting different phases: initiation, elongation and autophagosome formation, fusion, and autolysosome formation. Interestingly, treatment of K562 leukemic cells with DNR increased the expression of *GABARAPL1, ATG14,* and *SMPD1* without changing *miR-15a-5p* expression, suggesting that DNR regulates autophagy through another mechanism that remains to be elucidated. We also observed a negative correlation between *miR-15a-5p* and autophagy gene expression in chemosensitive and chemoresistant AML patients, suggesting the implication of autophagy regulation by *miR-15a-5p* in chemoresistance.

Altogether, our studies revealed that *miR-15a-5p* might play a dual role in the emergence of drug resistance to DNR by regulating both apoptosis and autophagy. *MiR-15a-5p* could be considered as a poor predictive biomarker in AML. Further clinical studies would be required to analyze whether patients with a higher level of expression of *miR-15a-5p* would benefit from DNR dose intensification. In addition, our results suggested that inhibiting *miR-15a-5p* could be a promising adjuvant therapeutic option in drug-resistant AML patients. Indeed, the modulation of *miR-15a-5p*, using antagomiRs for instance, could restore key genes involved in autophagy and in apoptosis and improve remission rates mainly in chemoresistant AML patients [37].

## 4. Materials and Methods

### 4.1. Cell Culture

Human embryonic kidney (HEK)-293T cells (obtained from ATCC, Manassas, VA, USA) were cultured in Dulbecco’s Modified Eagle’s Medium (DMEM, Lonza, Basel, Switzerland) with 10% fetal bovine serum (FBS) and with 50 U/mL penicillin and 50 mg/mL streptomycin (Gibco, Life technologies, Grand Island, NY, USA). The human AML cell lines K562 and KG1a (purchased from DSMZ, Braunschweig, Germany) were cultured in RPMI 1640 medium (Lonza) supplemented with 10% or 20% FBS, respectively, and with 50 U/mL penicillin and 50 mg/mL streptomycin.

### 4.2. Reagents

Daunorubicin hydrochloride (#30450) and chloroquine diphosphate salt (#C6628) were purchased from Sigma Aldrich (Saint-Louis, MO, USA). Three-Methyladenine (#S2767) was from Selleckchem (Houston, TX, USA).

### 4.3. Cell Transient Transfection

The synthetic *miR*Vana^TM^ hsa-miR-15a-5p mimic (#MC10235) and hsa-miR-15a-5p inhibitor oligonucleotides (#MH10235) were purchased from Life technologies. A total of five million K562 or KG1a cells were nucleoporated using Amaxa^®^ Nucleofector^®^ Technology (Lonza) with 100 µl of solution V or solution L, respectively (program T-016 for K562 and V-001 for KG1a) and with 750 pmol of precursor oligonucleotide and cultured for 24 h or 48 h. Scrambled oligonucleotide, *miR*Vana^TM^ miRNA Mimic Negative Control #1 (#4464058), was used as control. For siRNA transfection, a total of five million K562 cells were nucleoporated using the same method with a combination of four ON-TARGETplus human siRNAs against four target genes (ATG9A: #L-014294-01, ATG14: #L-020438-01, GABARAPL1: #L-014715-00, and SMPD1: #L-006676-00, Dharmacon, Lafayette, CO, USA) at a concentration of 25 nM for each siRNA or with 100 nM of ON-TARGETplus Non-targeting pool (#D-001810-10, Dharmacon, Lafayette, CO, USA) as control.

### 4.4. Protein Extraction and Western Blotting

Medium was removed and cells were washed in cold PBS before lysis in buffer (25 mM Tris/HCl pH 7.4, 150 mM NaCl, 6 mM EDTA, 10% glycerol and 1% Triton X-100) containing protease inhibitors (1 mM Pefabloc^®^ and 1 µg/mL aprotinin). Cells were incubated on ice for 20 min. Extracts were cleared by centrifugation (10,000 *g* × 10 min at 4 °C), and protein concentration was determined using the BCA Protein Assay Kit (Thermo Fisher Scientific, Waltham, MA, USA). Protein extracts (30–40 µg) were loaded on 4–15% precast polyacrylamide gels (Mini-PROTEAN^®^ TGX™, BioRad, Hercules, CA, USA). Following SDS-PAGE, proteins were transferred onto polyvinylidene difluoride membranes, which were then blocked in 5% fat-free milk powder in PBS. The membranes were incubated overnight at 4 °C with the indicated primary antibodies and then washed extensively before and after incubation for 1 h with horseradish peroxidase-conjugated secondary antibodies (Cell Signaling Technology, Danvers, MA, USA). Anti-LC3B (#2775, CST), p62 (#5114, CST), ATG9a (#D409D, CST), ATG14 (#96752, CST), and anti-GABARAPL1 (#26632, CST) antibodies were used at a dilution of 1:1000. Anti-LAMP-2 antibody (#sc-18822, Santa Cruz Biotechnology, Dallas, TX, USA) and anti-β-actin (#A-5441, Sigma Aldrich, Saint-Louis, MO, USA) were used at dilutions of 1:500 and 1:5000, respectively. Immunodetection was performed using chemiluminescence (Western blot Luminol Reagent, Santa Cruz, supplemented with 20% SuperSignal^®^ West Femto, Thermo Fisher Scientific).

### 4.5. RNA Extraction and RT-qPCR

Total RNA was extracted using TriPure Isolation Reagent (Sigma Aldrich, Saint-Louis, MO, USA). For the quantification of miRNA expression levels, 50 ng of total RNA was used for reverse transcription using the TaqMan MicroRNA Reverse Transcription Kit (Life Technologies), and real-time PCR was performed in triplicate using the TaqMan MicroRNA assay kit (hsa-miR-15a-5p: ID000389, RNA-control RNU44: ID001094, Life Technologies) according to the instructions of the manufacturer. Normalization was completed with small nucleolar *RNU44,* and the relative expression was calculated using the comparative cross threshold (Ct) method. For the quantification of mRNA levels, 1 µg of total RNA was subjected to reverse transcription using MMLV (Moloney Murine Leukemia Virus) reverse transcriptase enzyme (Invitrogen, Carlsbad, CA, USA). Quantitative PCR analysis was performed in triplicate using ABgene SYBR green-based kits (Thermo Fisher Scientific) using the oligonucleotides shown in Table 1 as described previously [38,39]. The ribosomal protein *RPLP0* served as normalization control.

### 4.6. Immunofluorescence

Cells were transferred to slides by centrifugation at 250 rpm for 5 min according to the Cytospin method. Cells were then fixed using 4% paraformaldehyde (#P6148, Sigma Aldrich) in PBS at pH 7.4 at room temperature for 15 min, permeabilized by incubation with 0.05% saponin (#47036, Sigma Aldrich) in PBS pH 7.4 for 20 min, and blocked using Q-PBS solution (PBS + 1% BSA + 0.01% saponin) for 20 min. Cells were incubated overnight at 4 °C with the indicated primary antibodies in Q-PBS (dilution of 1:200) and then washed three times with Q-PBS before incubation at room temperature for 1 h with the secondary antibodies (Alexa Fluor^®^ 488 donkey anti-rabbit #A21206 or Alexa Fluor^®^ 568 goat anti-mouse #A11004 dilution 1:500 and DAPI dilution 1:5000, Invitrogen, Carlsbad, CA, USA). The stained cells were washed two times with Q-PBS and two times with PBS and then post-fixed using 4% paraformaldehyde in PBS at pH 7.4 at room temperature for 10 min. Cells were finally washed three times with PBS, and coverslips were added on top of Dako Fluorescence mounting medium (Agilent Technologies, Santa Clara, CA, USA).

### 4.7. Cell viability Assay

K562 cells were seeded in 96-well plates (10.000 cells/well) in 100 µL of serum-supplemented medium. After 72 h, 100 µL of the single reagent (CellTiter-Glo^®^ Reagent) was directly added and mixed into the cell culture. After 10 min, the luminescent signal was monitored using a GloMax^®^ instrument (Turner Biosystems, Sunnyvale, CA, USA).

### 4.8. Microarray Assay

Microarrays were performed according to the Affymetrix ^®^ WT PLUS standard protocol. In total, 100 ng of total RNA was used as a starting material. The GeneChip WT PLUS Reagent Kit (Affymetrix, Santa Clara, CA, USA) was used for ss-cDNA preparation, fragmentation, and labeling. Human Transcriptome Arrays 2.0 (HTA2.0) chips were used for hybridization. The hybridization, wash, and scan were performed according to the Affymetrix kits and procedures specific to the HTA2.0 chips. After the scan, the quality controls of the hybridization were checked using the Gene Expression Console software (Affymetrix, Santa Clara, CA, USA). The RMA-Sketch procedure was used for data normalization. Fold change was determined for each comparison based on these normalized values. We found 520 genes differentially expressed after *miR-15a-5p* overexpression in K562 cells compared to the scrambled condition, of which 369 genes were downregulated (Figure S1A) and 151 were upregulated. The list of the most downregulated mRNAs (with the highest negative fold change) that were predicted as target genes by TargetScan program is shown in Figure S2B. We used the DAVID web tool [40] for the functional annotation and pathway analysis of the selected gene list (Figure S2C).

### 4.9. Cloning and Site-Directed Mutagenesis

The 3′UTR segments containing the target sites for *miR-15a-5p* (TGCTGCT) were amplified by PCR from cDNA and inserted into a pMirTarget vector (#PS100062, OriGene) between the *EcoRI* and *NotI* restriction sites for *ATG9A* (ENST00000361242.9, +3387 to +3648 pb), *SMPD1* (ENST00000342245.9, +2108 to +2410 pb) and *GABARAPL1* (ENST00000266458.10, +731 to +1611 pb), and between the *MluI* and *NotI* restriction sites for *ATG14* (ENST00000247178.6, +1842 pb to +4666 pb). All the mutants were generated by the deletion of 5 bp within the site of perfect complementarity using the QuickChange™ XL-II kit (Stratagene, La Jolla, CA, USA) according to the manufacturer’s protocol. Mutagenesis primers were synthesized by Eurogentec (Ougrée, Belgium) and are listed in Table 2. All the constructs were verified by sequencing.

### 4.10. Luciferase Assays

HEK-293T cells were seeded in 12-well plates (2 × 10^5^ cells/well). After one day, cells were co-transfected by the calcium phosphate method as follows [41]. Luciferase construct containing the wild-type or mutated 3′UTR segment of target genes (pMIR-ATG9A, pMIR-ATG14, pMIR-GABARAPL1 or pMIR-SMPD1, 0.125 µg), pEF1-β-galactosidase (0.3 µg, Invitrogen) as internal control and scrambled oligonucleotide or synthetic miR-15a-5p mimic (100 pmol) were diluted in 100 µl of water and mixed with 45 µL of BBS buffer (50mM *N,N*-bis-(2-hydroxyethyl)-2-aminoethane-sulfonic acid at pH 7, 280 mM NaCl, 1.5 mM Na_2_HPO_4_) and 4.5 µL CaCl_2_ 2.5 M (final volume 200 µL). After 24 h, cells were lysed, and the luciferase activity was monitored using a GloMax^®^ instrument (Turner Biosystems, Sunnyvale, CA, USA) as described previously [42]. The β-galactosidase activity was assessed as described [43]. The data are presented as the average ratio between the luciferase and the β-galactosidase activities and normalized to the scrambled condition.

### 4.11. Gene Expression Profiles of Patient Samples

Frozen diagnostic bone marrow RNA samples were obtained from 38 adults who had a confirmed diagnosis of AML. Twenty-four of these patients were considered “chemosensitive” because they reached a complete response after receiving one cycle of induction chemotherapy. The 14 remaining patients were considered “chemoresistant”, defined as having more than 5% blast cells in the bone marrow after induction chemotherapy. Cytogenetic analyses and patient clinical characteristics were described in Table S1 in our previous paper [21]. Blasts and mononuclear cells at diagnosis were purified by Ficoll–Hypaque (Nygaard) density gradient centrifugation and cryopreserved. RNA was isolated with either RNA-Bee or RLT following the protocols of the manufacturer (Bio-Connect BV, Huissen, The Netherlands). Expression levels of the four target genes—*ATG9A* (202492_at), *ATG14* (204568_at, 233984_at), *GABARAPL1* (211458_s_at, 208868_s_at, 208869_s_at), and *SMPD1* (209420_s_at)—were analyzed in 38 AML patient samples by using Affymetrix U133A GeneChips HOVON dataset as previously described (GEO accession: GSE6891) [30].

### 4.12. Statistical Analysis

Experiments were repeated at least three times with identical results. In most figures, the average of multiple replicate experiments is shown with the standard error of the mean (SEM), unless otherwise stated. Statistical analysis was performed using a bilateral Student’s t-test, Wilcoxon test, correlation test or a two-way ANOVA followed by Bonferroni’s test (*, *p* < 0.05; **, *p* < 0.01; ***, *p* < 0.001) using GraphPadPrism7 software.

## Figures and Tables

**Table 1:** List of oligonucleotides for qPCR.

Gene	Oligonucleotide Forward	Oligonucleotide Reverse
*ATG9A*	CTCTGCTAGCTATCCCTGTGC	GCACTGTGCCAGGATCTGT
*ATG14*	GCGATGAAGAAACCGACCT	CACAAAACCGGGGACTAGG
*GABARAPL1*	TGGGCCAACTGTATGAGGA	CTACCCCAAGTCCAGGTG
*SMPD1*	TGGCTCTATGAAGCGATGG	TGGGGAAAGAGCATAGAACC
*RPLP0*	TCGACAATGGCAGCATCTAC	ATCCGTCTCCACAGACAAGG

**Table 2:** List of oligonucleotides for mutagenesis.

Mutant	Oligonucleotide
*ATG9A∆*	CCTGGGCCCTCATTTTATCGTACCCCCC
*ATG14∆1*	GGGGTGAGTTACACGTATTTTATTCATTCTGTCGTAGTTTGTCAG
*ATG14∆2*	TAAGTATACATTTCAACCACTGTTTTTTCTACTCTTTTTTCA-TTAAAATCTTTCATGTA
*GABARAPL1∆1*	TTTCACATGCTCAATTGATATTTTTTTTCCTCGGCCCAGG
*GABARAPL1∆2*	AGGATTCTTGCTCCCATTGTCCCTTCAGGCTC
*SMPD1∆*	TCAGGTCGCAAGTACAGGGTTCCTGGCTG

## Data Availability

Microarray data are available in the ArrayExpress database (Available online: http://www.ebi.ac.uk/arrayexpress, (accessed on 11 February 2021) [44]) under accession number E-MTAB-8776.

## Supplementary Materials

The following are available online at https://www.mdpi.com/article/10.3390/ijms22105153/s1.
