# Supplementary material for: MiR-15a-5p Confers Chemoresistance in Acute Myeloid Leukemia by Inhibiting Autophagy Induced by Daunorubicin
Source: Int J Mol Sci. 2021 May 13;22(10):5153. doi: 10.3390/ijms22105153 (PMC8152749; doi:10.3390/ijms22105153)
Supplement: Supplementary file 1 [file ijms-22-05153-s001.zip › ijms-1220572-supplementary.pdf]

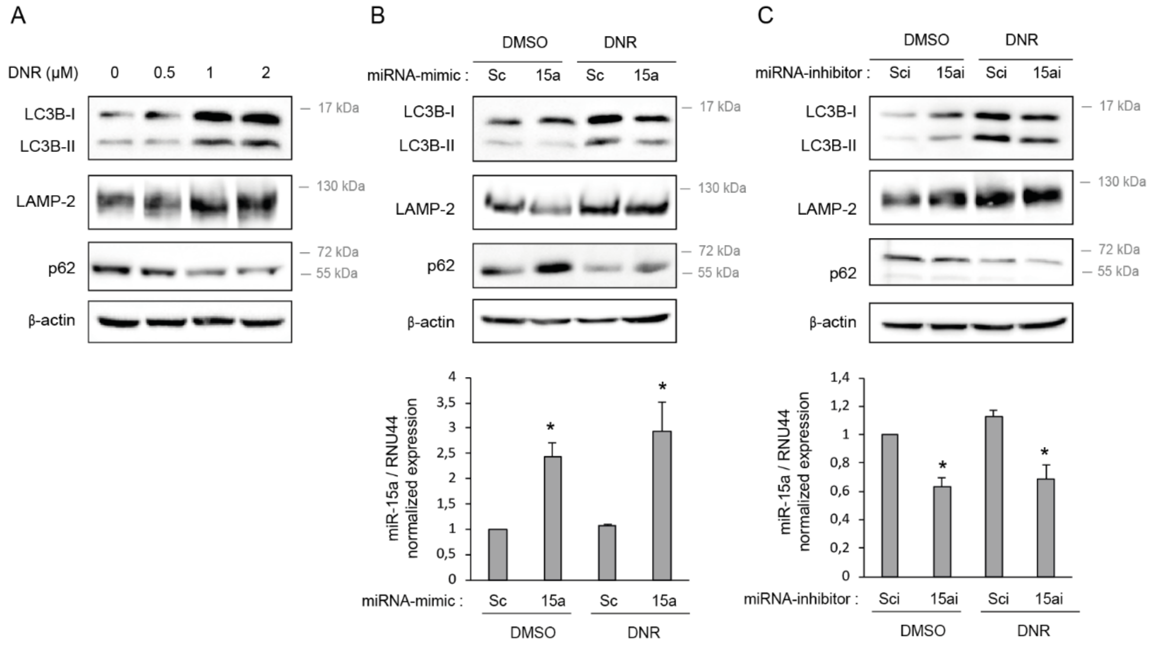

Figure S1. miR-15a-5p regulates autophagy induced by daunorubicin in KG1a cells. (A) KG1a cells were treated with different indicated doses of daunorubicin (DNR) or vehicle (DMSO) for 24 h. (B) KG1a cells were transiently transfected with miR-15a-5p mimic (15a) or scrambled mimic (Sc) as control for 48 h. (C) KG1a cells were transiently transfected with miR-15a-5p inhibitor (15ai) or scrambled inhibitor (Sci) as control for 48 h. (B-C) Cells were treated with 2μM of daunorubicin (DNR) for the last 24 h before protein and RNA extraction. (A-C) Level of autophagy was analyzed by western blotting with anti-LC3B, anti-LAMP-2, and anti-p62 antibodies. Expression of β-actin was also detected as a loading control. (B-C) miR-15a-5p expression was measured by RT-qPCR and normalized with the expression of RNU44. The scrambled condition was set to 1. The normalized means of three independent experiments are shown with SEM. (*, $p<0.05$, compared with the scrambled condition).

A

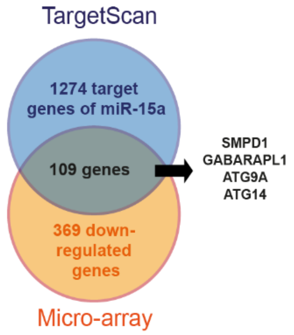

B

Probe Set ID	miR-15a vs scramble 1 (logFC)	miR-15a vs scramble 2 (logFC)	Gene Symbol	Gene Description
TC11003457.hg.1	-1,205423	-1,104408	ARL2	ADP-ribosylation factor-like 2
TC11000131.hg.1	-1,166028	-0,438861	SMPD1	sphingomyelin phosphodiesterase 1, acid lysosomal
TC12000159.hg.1	-1,145463	-0,601323	GABARAPL1	GABA(A) receptor-associated protein like 1
TC01001685.hg.1	-0,888098	-0,028212	BTG2	BTG family, member 2
TC10000808.hg.1	-0,843751	-0,268	PDCD4	programmed cell death 4
TC19000416.hg.1	-0,814478	-0,343602	CCNE1	cyclin E1
TC17000349.hg.1	-0,681441	-0,234493	CPD	carboxypeptidase D
TC03001374.hg.1	-0,621196	-0,311853	CDC25A	cell division cycle 25A
TC14001159.hg.1	-0,575486	-0,027263	ATG14	autophagy related 14
TC02005055.hg.1	-0,566423	-0,298785	ATG9A	autophagy related 9A
TC18000132.hg.1	-0,503195	-0,340869	RNF125	ring finger protein 125, E3 ubiquitin protein ligase
TC01000302.hg.1	-0,475222	-0,386172	LYPLA2	lysophospholipase II
TC04000689.hg.1	-0,468351	-0,226494	SCOC	short coiled-coil protein

C

Annotation Cluster 9	Enrichment Score: 1.32	G		Count	P_Value	Benjamini
UP_KEYWORDS	Autophagy	RT		5	4.8E-3	5.2E-2
GOTERM_CC_DIRECT	autophagosome membrane	RT		3	8.9E-3	3.2E-1
GOTERM_BP_DIRECT	autophagosome assembly	RT		3	2.7E-2	9.1E-1
UP_KEYWORDS	Cytoplasmic vesicle	RT		4	4.7E-1	1.0E0
UP_KEYWORDS	Endoplasmic reticulum	RT		7	4.8E-1	1.0E0

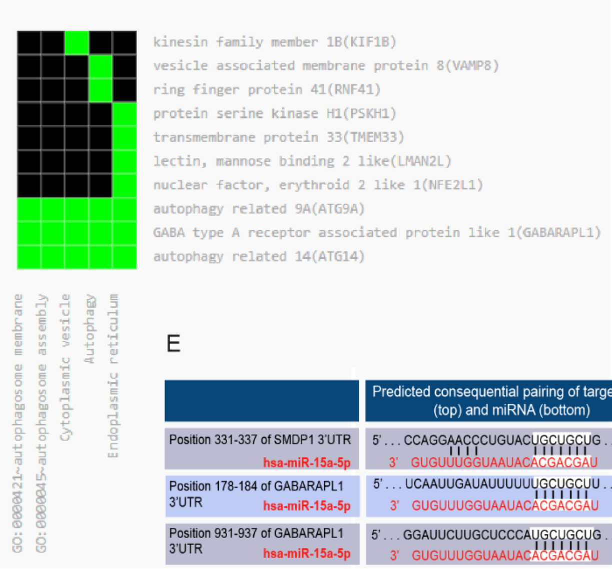

D

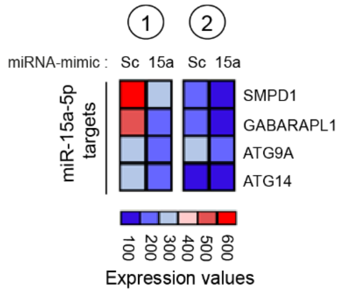

E

	Predicted consequential pairing of target region (top) and miRNA (bottom)	Site type	Context ++ score	Context++ score percentile	Weighted context++ score	Conserved branch lenght	PCT
Position 331-337 of SMDP1 3'UTR hsa-miR-15a-5p	5' . . . CCAGGAACCCUGUACUGCUGCUG . . .<br>3' GUGUUUGGUAAUACACGACGAU	7 mer-m8	-0.29	95	-0.29	4.625	0.69
Position 178-184 of GABARAPL1 3'UTR hsa-miR-15a-5p	5' . . . UCAAUUGAUAUUUUUUGCUGCUU . . .<br>3' GUGUUUGGUAAUACACGACGAU	7 mer-m8	-0.37	96	-0.37	2.880	0.44
Position 931-937 of GABARAPL1 3'UTR hsa-miR-15a-5p	5' . . . GGAUUCUUGCUCCCAUGCUGCUG . . .<br>3' GUGUUUGGUAAUACACGACGAU	7 mer-m8	-0.12	53	-0.12	0.379	<0.1
Position 750-756 of ATG9A 3'UTR hsa-miR-15a-5p	5' . . . UUCCUGGGCCCUCAUUGCUGCUU . . .<br>3' GUGUUUGGUAAUACACGACGAU	7 mer-m8	-0.33	97	-0.33	3.554	0.56
Position 571-577 of ATG14 3'UTR hsa-miR-15a-5p	5' . . . CACGUAUUUUAUUCAUGCUGCUC . . .<br>3' GUGUUUGGUAAUACACGACGAU	7 mer-m8	-0.20	87	-0.04	4.336	0.66
Position 2753-2759 of ATG14 3'UTR hsa-miR-15a-5p	5' . . . UUUUUCUACUCUUUUUGCUGCUC . . .<br>3' GUGUUUGGUAAUACACGACGAU	7 mer-m8	-0.28	94	-0.05	4.923	0.71

Figure S2. Predicted autophagy target genes of miR-15a-5p. K562 cells were transiently transfected with miR-15a-5p mimic or scrambled mimic as control and simultaneously treated with 1 μM of daunorubicin for 24 h. RNA were extracted and gene expression profiles (Affymetrix) were performed in duplicate using microarray assays. (A) The list of downregulated mRNAs was crossed with target genes identified by the bioinformatics prediction program TargetScan and several genes involved in autophagy were identified. (B) The most important downregulated genes and their expression were resumed in the table. (C) DAVID web tool was used for pathway analysis of the selected gene list and revealed several functional clusters including autophagy. (D) Expression change of predicted autophagy target genes was shown for the two replicate experiments. (E) Predicted binding sites, identified by TargetScan, for miR-15a-5p in the 3'UTR region of genes involved in autophagy are resumed in the table.
